# IRF2 maintains the stemness of colonic stem cells by limiting physiological stress from interferon

**DOI:** 10.1038/s41598-020-71633-3

**Published:** 2020-09-08

**Authors:** Kana Minamide, Taku Sato, Yusuke Nakanishi, Hiroshi Ohno, Tamotsu Kato, Jumpei Asano, Toshiaki Ohteki

**Affiliations:** 1 Department of Biodefense Research, Medical Research Institute, Tokyo Medical and Dental University (TMDU), 1-5-45 Yushima, Bunkyo-ku, Tokyo, 113-8510 Japan; 2 Precursory Research for Embryonic Science and Technology (PRESTO), Japan Science and Technology Agency, Tokyo, Japan; 3 Laboratory for Intestinal Ecosystem, RIKEN Center for Integrative Medical Sciences (IMS), Kanagawa, Japan

**Keywords:** Mechanisms of disease, Intestinal stem cells

## Abstract

The physiological stresses that diminish tissue stem-cell characteristics remain largely unknown. We previously reported that type I interferon (IFN), which is essential for host antiviral responses, is a physiological stressor for hematopoietic stem cells (HSCs) and small intestinal stem cells (ISCs) and that interferon regulatory factor-2 (IRF2), which attenuates IFN signaling, maintains their stemness. Here, using a dextran sodium sulfate (DSS)-induced colitis model, we explore the role of IRF2 in maintaining colonic epithelial stem cells (CoSCs). In mice with a conditional *Irf2* deletion in the intestinal epithelium (hereafter *Irf2*^*ΔIEC*^ mice)_,_ both the number and the organoid-forming potential of CoSCs were markedly reduced. Consistent with this finding, the ability of *Irf2*^*ΔIEC*^ mice to regenerate colon epithelium after inducing colitis was severely impaired, independently of microbial dysbiosis. Mechanistically, CoSCs differentiated prematurely into transit-amplifying (TA) cells in *Irf2*^*ΔIEC*^ mice, which might explain their low CoSC counts. A similar phenotype was induced in wild-type mice by repeated injections of low doses of poly(I:C), which induces type I IFN. Collectively, we demonstrated that chronic IFN signaling physiologically stresses CoSCs. This study provides new insight into the development of colitis and molecular mechanisms that maintain functional CoSCs throughout life.

## Introduction

The colonic epithelial layer acts as a physical and biological barrier against luminal commensal bacteria and intestinal pathogens. Thus, colonic epithelial integrity must be tightly regulated to prevent intestinal inflammation triggered by commensal bacterial translocation. Breakdown of the colonic epithelial layer allows the entry of commensal flora, which induces improper immune responses against the commensal flora, leading to inflammatory bowel disease (IBD) development.

Colonic stem cells (CoSCs) at the base of the intestinal crypts are essential for the constitutive turnover of the entire epithelium at steady state and for epithelial regeneration after injury. Recent research found that leucine-rich G protein-coupled receptor 5 (Lgr5) is specifically expressed in stem cells in both the small intestine and colon^1^. Genetic lineage-tracing studies in vivo and organoid formation assays ex vivo suggested that *Lrig1, Ephb2*, *Sox9*, and *Krt19* also serve as physiological CoSC markers^2–5^. A special microenvironment that supports stem-cell properties is referred to as a stem-cell niche. Niche-derived factors such as Wnt, Notch, and EGF are critical for maintaining intestinal stem cells^6^. However, the environmental stresses that impair stemness, and the systems that protect stemness against such stressors, remain to be elucidated.

Type I interferons (IFNs) are key cytokines that stimulate intracellular responses against viral infection, such as the degradation of viral DNA or RNA^7,8^. The transcription factor interferon regulatory factor 2 (IRF2) negatively regulates type I IFN signaling^9^. Given that type I IFNs are produced constitutively at low levels, and IRF2 is expressed constitutively by a variety of cell types and tissues under specific pathogen-free and steady-state conditions^10,11^, we hypothesized that IRF2 perpetually protects epithelial stem cells from physiological damage due to excessive IFN. Notably, our group and others previously showed that type I IFNs attenuate the functions of hematopoietic stem cells (HSCs) in mouse bone marrow^12,13^. In addition, we have recently showed that regulated IFN-signaling preserves the stemness of small intestinal stem cells (ISCs) by restricting differentiation into secretory-cells^14^.

In this study, we found that deleting *Irf2* specifically in the intestinal epithelium of mice reduced the number of CoSCs and severely impaired epithelial regeneration after the development of DSS-induced colitis. We also confirmed that the organoid-forming potential of CoSCs in wild-type (WT) mice was substantially reduced by ongoing treatment with low doses of poly(I:C), which induces type I IFN in vivo, indicating that chronic IFN signaling causes CoSC function to decline. Therefore, IRF2 plays a critical role in preserving the stemness of CoSCs by suppressing physiological IFN signaling.

## Results

### Regeneration of the colonic epithelium is defective in *Irf2*^***ΔIEC***^ mice

**Figure 1:**
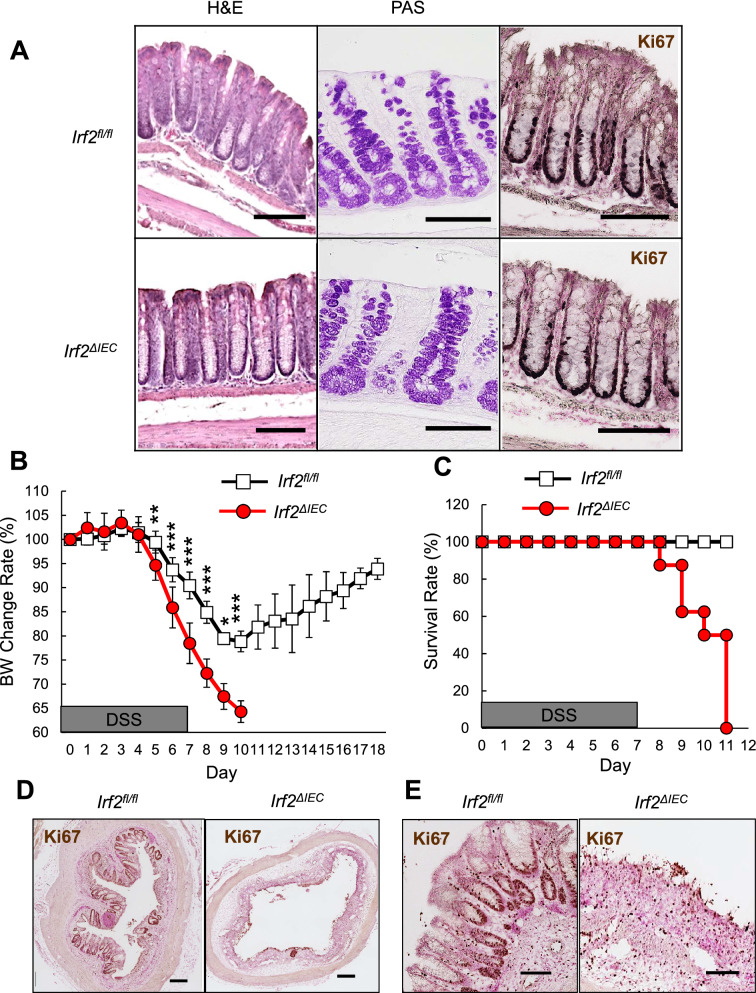
Defective regeneration of colonic epithelium in *Irf2*^*ΔIEC*^ mice. (**A**) H&E (left) and PAS staining (middle) of distal colon tissue from *Irf2*^*fl/fl*^ and *Irf2*^*ΔIEC*^ mice (n = 3). Proliferating cells were detected by Ki67 staining (right). Scale bars: 100 μm. (**B**,**C**) Graphs show changes in body weight (BW) and mortality over time between *Irf2*^*fl/fl*^ and *Irf2*^*ΔIEC*^ mice that were given 2% DSS (w/v) in drinking water for 7 days followed by 11 days of plain water (n = 8 per group). (**D**) Low-magnification and (**E**) high-magnification images show histologic analysis of the distal colon in *Irf2*^*fl/fl*^ and *Irf2*^*ΔIEC*^ mice treated with 2% DSS in drinking water for 5 days followed by 6 days of plain water (n = 3). Regenerating crypts were detected by Ki67 staining. Scale bars: (D) 200 μm and (E) 100 μm. *P < 0.05, **P < 0.01, ***P < 0.001 by Student's *t* test.

To examine the role of IRF2 in the homeostasis and regeneration of colonic epithelial cells after injury, we crossed *Irf2-*floxed mice with *Villin-Cre* mice to generate mice deficient in *Irf2* specifically in the intestinal epithelial cells (hereafter *Irf2*^*ΔIEC*^ mice). In the steady state, *Irf2*^*ΔIEC*^ mice had no obvious abnormalities in the colonic epithelial layer, including the crypt shape and the size, the development and distribution of goblet cells, and the number of Ki67^+^ proliferating cells, indicating that IRF2 is dispensable for the development and physiologic turnover of the colonic epithelium (Fig. 1A). We next examined the colonic epithelium’s regenerative capacity in *Irf2*^*ΔIEC*^ mice. To this end, *Irf2*^*ΔIEC*^ and littermate control *Irf2*^*fl/fl*^ mice (hereafter, control mice) were treated with DSS to induce colitis. Notably, 7 days of DSS treatment induced a greater loss of body weight and significantly shortened survival in *Irf2*^*ΔIEC*^ mice compared with control mice (Fig. 1B,C). Histologically, the Ki67^+^ proliferating cells and crypt structure were largely restored in control mice but remained impaired in *Irf2*^*ΔIEC*^ mice 6 days after DSS treatment ended (Fig. 1D,E). These findings indicated that IRF2 is important for preserving the capacity of the colon epithelium to regenerate after injury.

### IRF2 deficiency does not cause dysbiosis or disrupt the mucus layer

**Figure 2:**
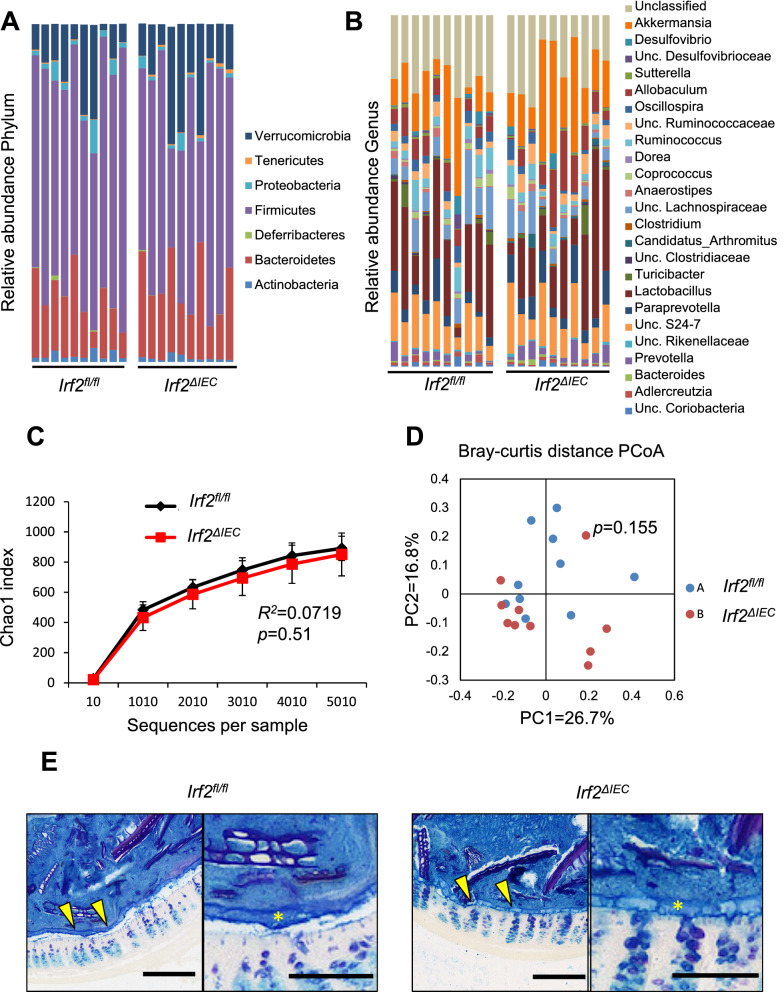
IRF2 deficiency does not affect the composition of commensal bacteria or the mucus-layer structure. (**A**, **B**) 16S rRNA gene-sequencing analysis showing the relative abundance of bacteria identified at the phylum (**A**) and genus (**B**) level in fecal samples of co-housed *Irf2*^*fl/fl*^ and *Irf2*^*ΔIEC*^ mice (n = 10). At the genus level, the sequences from the samples represented 106 genera; the top 25 are listed in (**B**). Sequences that could not be classified into any known genus are shown as unclassified (Unc). (**C**) Rarefaction curves of bacterial alpha diversity using the Chao1 index. Significance was calculated by a non-parametric two-sample *t* test. (**D**) Principal coordinates analysis (PCoA) of beta diversity using Bray–Curtis distances. Significance was calculated by PERMANOVA. (**E**) Representative images of Alcian blue/PAS-stained distal colon sections of *Irf2*^*fl/fl*^ and *Irf2*^*ΔIEC*^ mice (n = 3). Arrowheads indicate the mucus layer. Asterisks indicate the area of mucus layer. Scale bars: 200 μm (left panels, low-magnification) and 100 μm (right panels, high-magnification).

Dysbiosis, which is an altered composition of intestinal microflora, is involved in the pathogenesis of inflammatory bowel disease (IBD) in humans and in mouse colitis models^15,16^. To determine whether dysbiosis caused the increased susceptibility to DSS-induced colitis in *Irf2*^*ΔIEC*^ mice, we used 16S ribosomal RNA sequencing to survey the bacterial populations in fecal samples from naïve *Irf2*^*ΔIEC*^ mice and co-housed control littermates. The composition of microbiota at both the phylum and genus levels appeared similar between the control and *Irf2*^*ΔIEC*^ mice (Fig. 2A,B). Indeed, the alpha diversity, which represents the microbial diversity within individual samples, did not differ significantly between control and *Irf2*^*ΔIEC*^ mice (non-parametric two-sample *t* test, *P* = 0.51; Fig. 2C). Furthermore, the beta diversity, which represents the microbial diversity between samples and is shown as the Bray–Curtis distance, was also similar at the phylum level (PERMANOVA, *P* = 0.155; Fig. 2D).

Another possible cause for the enhanced susceptibility to colitis in *Irf2*^*ΔIEC*^ mice was dysfunction of the mucosal barrier, which would allow commensal bacteria to invade the intestinal mucosa (e.g., the epithelial layers and lamina propria)^17^. Thus, we examined the integrity of the mucus layer by staining colon tissues from control and *Irf2*^*ΔIEC*^ mice with Alcian blue/PAS, and found that the thickness of the mucus gel layer was comparable in these mice (Fig. 2E). We also checked the stability of the tight junctions by ZO-1 staining and found that it was also unchanged in the colon of *Irf2*^*ΔIEC*^ mice (Fig. S1). Collectively, these results indicated that the enhanced susceptibility of *Irf2*^*ΔIEC*^ mice to DSS-induced colitis was not due to dysbiosis or mucus-barrier dysfunction.

### IRF2 is essential for maintaining CoSCs

**Figure 3:**
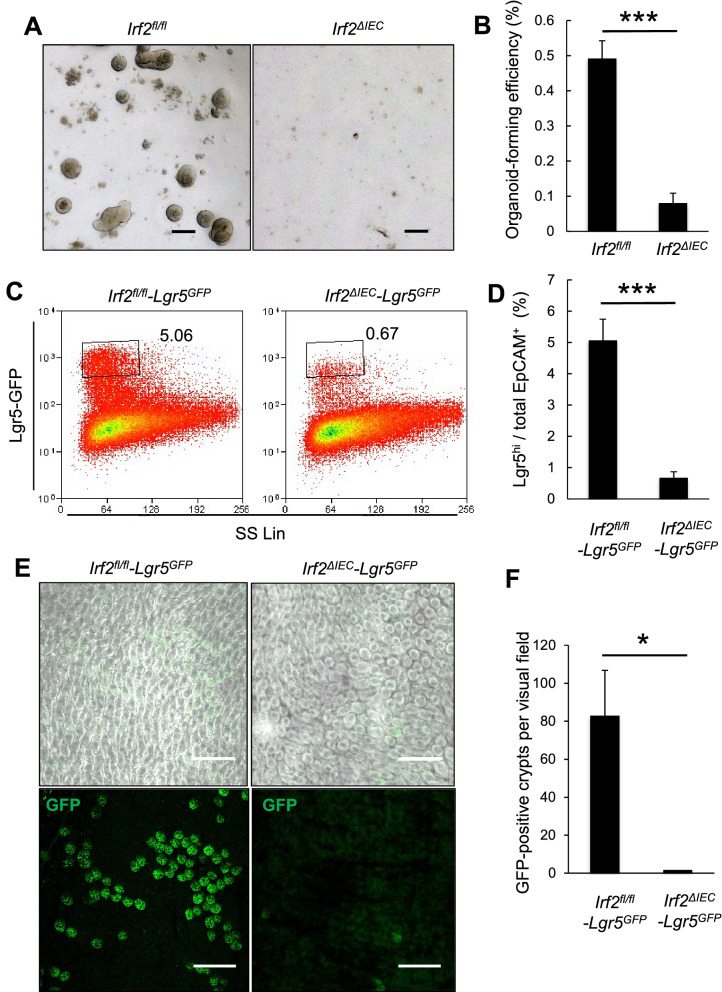
IRF2 is essential for maintaining CoSCs. (**A**) Representative images show organoids formed from single colonic EpCAM^+^ cells from *Irf2*^*fl/fl*^ and *Irf2*^*ΔIEC*^ mice (n = 4). Scale bars: 100 μm. (**B**) Organoid-formation efficiency was determined by counting the viable organoids in each well on day 6; data represent the mean ± SD for five independent experiments. ***P < 0.001 by Student’s *t* test. (**C**) Representative FACS plots for Lgr5-GFP^hi^ cells in colonic epithelial cells isolated from naïve *Irf2*^*fl/fl*^*-Lgr5*^*GFP*^ and *Irf2*^*ΔIEC*^*-Lgr5*^*GFP*^ mice (n = 3 each). (**D**) Average percentage of Lgr5-GFP^hi^ cells among EpCAM^+^ cells; data represent mean ± SD for 3 mice. ***P < 0.001 by Student’s *t* test. (**E**) Confocal images of the base of crypts in the colon of control *Irf2*^*fl/fl*^*-Lgr5*^*GFP*^ and *Irf2*^*ΔIEC*^*-Lgr5*^*GFP*^ mice (n = 3 each), showing merged fluorescent and phase-contrast images (upper panels) and fluorescent images (lower panels). Scale bars: 200 μm. (**F**) Number of GFP-positive crypts per visual field. Five fields of view were counted per mouse. Data represent mean ± SD for 3 mice. *P < 0.05 by Student’s *t* test.

CoSCs are found at the crypt bottom, where their survival and maintenance is supported by neighboring secretory cells^18^. CoSCs can be visualized in *Lgr5-EGFP-IRES-CreERT2* mice^1^. We observed *Irf2* mRNA expression in both the colon epithelial cells and Lgr5-positive CoSCs from these mice (Fig. S2). To test whether epithelial regeneration in the colon of *Irf2*^*ΔIEC*^ mice was impaired by a decrease in CoSC population or function, we directly evaluated stem-cell potential by a organoid-forming assay, which recapitulates the tissue-regenerative potential of CoSCs^19^. CoSCs and other colonic epithelial cells isolated from *Irf2*^*ΔIEC*^ and control mice by flow cytometry were seeded in Matrigel containing appropriate growth factors. On day 6 of culture, we counted the number of organoids, defined as viable sphere structures consisting of a single epithelial layer with a lumen. Crypt epithelial cells from *Irf2*^*ΔIEC*^ mice had a significantly lower organoid-forming efficiency than those from control mice (Fig. 3A,B), indicating that IRF2 is essential for preserving the CoSCs’ regenerative potential. To determine whether IRF2 deletion affects the number of CoSCs, we crossed *Lgr5-EGFP-IRES-CreERT2* mice (hereafter *Lgr5*^*GFP*^ mice) with *Irf2*^*ΔIEC*^ mice to visualize CoSCs in the *Irf2*^*ΔIEC*^ mice. Flow cytometry analysis showed significantly fewer Lgr5^hi^ cells in *Irf2*^*ΔIEC*^*-Lgr5*^*GFP*^ mice than in *Lgr5*^*GFP*^ control mice (Fig. 3C,D). We confirmed by confocal microscopy that the number of crypts containing Lgr5-positive CoSCs was decreased in the *Irf2*^*ΔIEC*^*-Lgr5*^*GFP*^ mice (Fig. 3E,F). Given that the number of TUNEL + apoptotic cells in the colonic crypt was unaffected by the Irf2 deficiency (Fig. S3), it is likely that the IRF2 deletion reduces the self-renewal capacity of CoSCs. These results collectively suggested that IRF2 plays an essential role in epithelial regeneration in the colon by preserving CoSCs.

### Loss of IRF2 promotes cell cycle and differentiation of CoSCs

To identify the differentially regulated biological process that accounts for the reduction of CoSCs in *Irf2*^*ΔIEC*^ mice, we applied GSEA combined with the Molecular Signatures Database (MSigDB) to microarray data obtained from colonic crypt epithelial cells from naïve *Irf2*^*ΔIEC*^ and control mice. The gene set related to organ morphogenesis was significantly downregulated in *Irf2*^*ΔIEC*^ colonic epithelial cells (Fig. S4), suggesting that the epithelial cell regeneration after injury was dysregulated. Wnt and Notch signals are required for the maintenance of ISCs^20,21^. However, we did not find any attenuation of the signatures for Wnt-β catenin and notch signaling, which were somewhat up-regulated in the *Irf2*^*ΔIEC*^ mice (Fig. S4). The expression of representative genes from these pathways, such as Axin2, CD44, and Hes1, was unaltered in the colon of *Irf2*^*ΔIEC*^ mice compared with control *Irf2*^*fl/fl*^ mice. These results suggested that IRF2 maintains the CoSCs independently of Wnt and Notch signaling.

**Figure 4:**
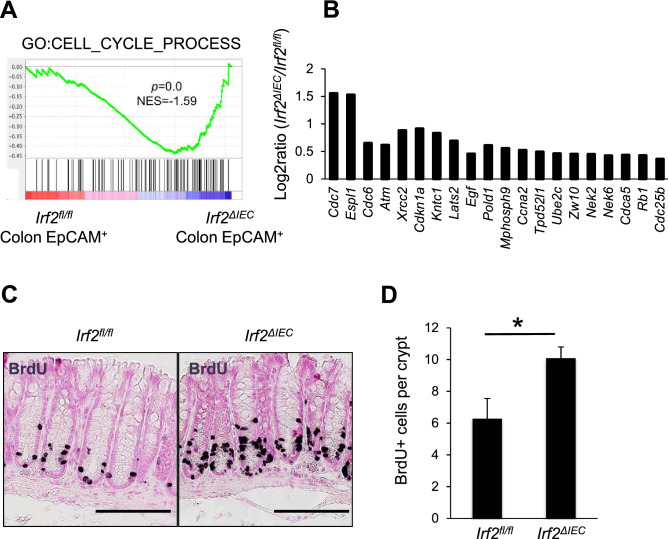
Loss of IRF2 promotes cell cycle and differentiation of CoSCs. (**A**) GSEA for colonic epithelial cells from *Irf2*^*fl/fl*^ and *Irf2*^*ΔIEC*^ mice (n = 3 for each group) using C5 MSigDB gene sets (C5: GO gene sets). The cell cycle process was significantly enriched in *Irf2*^*ΔIEC*^ compared to *Irf2*^*fl/fl*^ mice. NES, normalized enrichment score. (**B**) The top 20 most up-regulated genes in the gene set for cell-cycle processes in *Irf2*^*ΔIEC*^ compared to *Irf2*^*fl/fl*^ mice. Results are depicted as the log2 ratio determined by microarray. (**C**) Representative images of BrdU staining of the colon crypts of *Irf2*^*fl/fl*^ and *Irf2*^*ΔIEC*^ mice (n = 3). Mice were sacrificed 2 h after BrdU injection. Scale bars: 100 μm. (**D**) Number of BrdU^+^ cells per crypt. Approximately 100 crypts per mouse were analyzed. Data represent the means ± SD for 3 mice. *P < 0.05 by Student’s *t* test.

Notably, the cell-cycle process gene signature was significantly enriched in the *Irf2*^*ΔIEC*^ crypt epithelial cells (Fig. 4A,B), suggesting that cell division was accelerated in these cells. Thus, we performed a short-term 5-bromo-2′-deoxyuridine (BrdU) single-pulse experiment to label cells in S-phase in the colon (Fig. 4C,D). Two hours after BrdU injection, the number of BrdU-incorporating colonic crypt cells in the *Irf2*^*ΔIEC*^ mice was significantly higher than that in control mice. These results strongly suggested that cell-cycle progression is accelerated in the colon of *Irf2*^*ΔIEC*^ mice, which might cause CoSCs to differentiate into transit-amplifying (TA) cells prematurely. A similar phenomenon was reported in intestinal SCs in mice deficient for *Yin Yang* 1 (*Yy1*), a zinc-finger transcriptional factor^22^, in which an accelerated commitment of Lgr5 + intestinal stem cells to differentiated populations causes their depletion.

### Chronic type I IFN signaling impairs CoSC function

**Figure 5:**
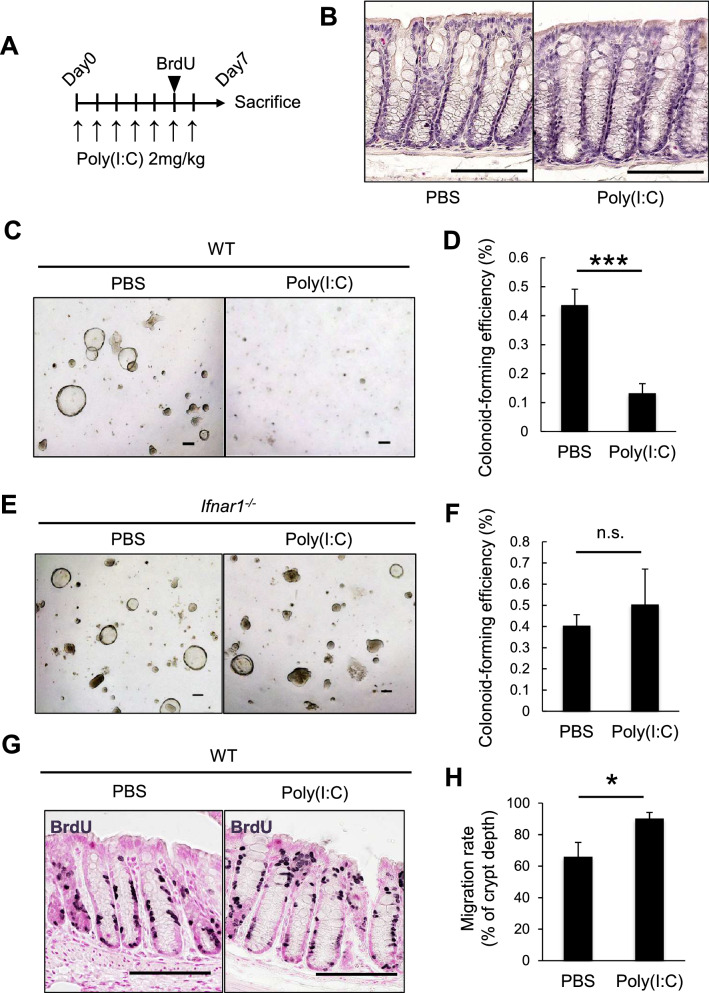
Chronic type I IFN signaling diminishes colonic epithelial stem-cell function. (**A**) Experimental protocol for poly(I:C) treatment. (**B**) H&E staining of the distal colon after 7 days of PBS or poly(I:C) treatment. Scale bars: 100 μm. (**C**, **D**) Representative images showing the organoid-forming efficiency of CoSCs from WT and (**E**,**F**) *Ifnar1*^*−/−*^ mice treated with either poly (I:C) or PBS as in (A) (n = 3 for each group). Scale bars: 50 μm. Data represent mean ± SD of five independent experiments. ***P < 0.001 by Student’s *t* test. n.s., not significant. (**G**) Representative images of the 2-day BrdU-tracing experiment of the colon of WT mice treated with either PBS or poly(I:C). As shown in (**A**), BrdU was injected into mice on day 5 of poly(I:C) treatment. Mice were sacrificed on day 7. Scale bars: 100 μm. (**H**) Migration rate of BrdU-labeled cells 2 days after BrdU injection. The distance of the BrdU-labeled cells from the bottom of the crypt was divided by the total depth of the crypt. At least 30 crypts per mouse were analyzed. Data represent mean ± SD for 3 mice. *P<0.05 by Student's *t* test.

Since IRF2 is a transcriptional attenuator of type I IFN signaling^11^, we next investigated whether increased type I IFN signaling affects the CoSC function in WT mice. Type I IFN is physiologically produced in the gastrointestinal tract by immune cells in response to commensal bacteria^23^. In mice treated with poly(I:C), a synthetic analog of double-strand RNA (dsRNA) and inducer of type I IFN, qPCR showed that the expression of typical IFN-inducible genes, such as *Ifit2*, *Ifitm3*, *Oas1g*, and *Stat1*, was up-regulated in the colonic epithelial cells of WT mice but was impaired in those of *Ifnar1*^*-/-*^ mice, which lack type I IFN receptors (Fig. S5). Based on background information and our experimental data, we established a protocol in which a low dose (2 mg/kg body weight) of poly(I:C) was injected for 7 consecutive days into WT mice; this treatment did not cause abnormalities in the colonic epithelium in the steady state (Fig. 5A,B).

We conducted an ex vivo organoid-forming assay to determine whether chronic type I IFN signaling affects CoSC function, and found that the organoid-forming efficiency of crypt epithelial cells was significantly reduced in poly(I:C)-treated compared to PBS-treated (control) WT mice (Fig. 5C,D). In sharp contrast, this functional decline was not observed in CoSCs from poly(I:C)-treated *Ifnar1*^*−/−*^ mice (Fig. 5E,F). These findings suggested that chronic excessive type I IFN signaling impairs the stemness of CoSCs.

To further investigate the effect of type I IFN signaling on colonic crypt cells, we performed a tracing experiment using BrdU labeling. In this method, the migration level of BrdU-labeled cells reflects the activity of CoSC division as it pushes older cells toward the apical lumen^24^. Comparison of the positions of BrdU-labeled cells 2 and 48 h after BrdU injection revealed that the migration distance of BrdU-labeled cells in poly(I:C)-treated mice was significantly longer than that in control PBS-treated mice (Figs. 5G,H and S6). These results suggested that CoSC division is enhanced upon IFN signaling, and further implied that the accelerated cell cycle of CoSCs might cause their exhaustion. Our data are consistent with a previous study showing that IFN signaling promotes the turnover of intestinal epithelia in the small intestine^25^.

## Discussion

In the present study, we demonstrated that IRF2 plays a critical role in preserving function and homeostasis of CoSC, major players in intestinal crypt regeneration^26,27^, by limiting the physiological IFN signaling, which is important for “revving” up the host immune system^10^. Mice with a conditional *Irf2* deletion in the intestinal epithelium, i.e. *Irf2*^*ΔIEC*^ mice, showed a clear decrease in both the number and the organoid-forming potential of CoSCs. In line with these CoSC phenotypes, the ability of *Irf2*^*ΔIEC*^ mice to regenerate the colonic epithelium after the development of DSS-induced colitis was severely impaired in a manner independent of microbial dysbiosis. Gene expression profiles of colonic crypt cells from *Irf2*^*ΔIEC*^ mice showed an upregulated cell cycle, supporting the scenario that the impaired self-renewal capacity of *Irf2*^*ΔIEC*^ CoSCs promotes their differentiation into TA cells.

The physiological relevance of IFN signaling to tissue stem-cell function is well studied in HSCs, in which type I IFNs directly induce proliferation^12,13^. A subsequent study showed that a systemic *Mycobacterium avium* infection upregulates intracellular IFN signaling and depletes HSCs^28^. These effects of IFNs on HSCs are considered to be one cause of pancytopenia, a secondary and common complication during infection and autoimmune diseases^29^. We previously and recently reported that uncontrolled physiological IFN signaling reduces the self-renewal capacity of HSCs and ISCs in *Irf2*^*−/−*^ mice, promoting their forced differentiation into hematopoietic progenitors and secretory progenitors, respectively^13,14^. IFNs promote host defense on one hand. Exogenous type I IFN signaling during viral infection promotes epithelial turnover in the kidney, small intestine, and salivary gland by activating the ERK pathway^25^. The modulation of epithelial migration and proliferation by infection-induced transient IFN signaling should improve mending after tissue injury and the exclusion of infected cells. On the other hand, sustained IFN signaling stresses create cellular stress and diminish self-renewal capacity of HSCs, ISCs and CoSCs in both physiological and pathological contexts. These findings further imply that IFNs may exert similar effects on a wide variety of tissue stem cells in mice and humans.

Recent reports indicate that in addition to IFNs, tissue stem cell microenvironments constitutively produce a variety of other physiological stressors, some which increase with age. In a wide range of species, neurogenesis declines dramatically with aging, and this decline is well correlated with reductions in the number of neural stem cells (NSCs) and the proliferative capacity of neural progenitors. In this context, CC-chemokine ligand 11 (Ccl11) is elevated in the plasma of aged mice, and CCL11 treatment decreases neurogenesis in young mice, indicating that the aging-related increase in systemic CCL11 causes NSC dysfunction^30,31^. In muscle fibers, FGF2 production is elevated with aging, which diminishes the function of muscle stem cells (MuSCs)^32^. Furthermore, MuSCs express Sprouty1, a negative regulator of FGF signaling, thereby inhibiting the effects of niche-derived FGF and preserving the stemness of MuSCs during aging. Accordingly, the functional relationship between Sprouty1 and FGF2 in MuSCs is remarkably similar to that between IRF2 and IFN in CoSCs. Although the mechanisms that protect tissue stem cells from stressors in their microenvironment are largely unknown, it seems likely that there are many strategies to reduce these physiologic stresses, given the irreplaceable role of tissue stem cells in maintaining the body.

Given that type I IFNs has been used for the treatment of viral hepatitis and multiple sclerosis, and some patients suffer from ulcerative colitis (UC) and celiac disease during type I IFN treatment^33,34^ the chronic IFN treatment may induce weakening of the IECs themselves by reducing stemness in addition to excessive activation of the immune system, leading to the development of these diseases.

## Methods

### Mice and ethics statement

We obtained C57BL/6J (B6) mice from Japan SLC, Inc.; *Villin-Cre* transgenic mice, *Lgr5-EGFP-Ires-CreERT2* (*Lgr5*^*ki*^) from Jackson Laboratory; *Irf2-flox* mice were generated as recently described^14^ For poly(I:C) (polyinosinic:polycytidylic acid) treatment, mice were injected intraperitoneally with PBS or 2 mg/kg body weight of poly(I:C) for 7 consecutive days. To induce mucosal injury in the colon, mice were given 2% w/v DSS (MP Biochemical, Santa Ana, CA) in drinking water for 5–7 days. DSS-treated mice were weighed daily. All mice were maintained in our SPF animal facility and were used for experiments at 3–4 months of age. All experiments using mice were approved by the Institutional Animal Care Committee of Tokyo Medical and Dental University and were performed in accordance with Tokyo Medical and Dental University guidelines.

### Histological analysis

The distal half of the colon tissue was harvested, fixed with 10% (w/v) Formaldehyde Neutral Buffer Solution (Nacalai, Kyoto, Japan) overnight, embedded in paraffin, sectioned at 5 μm, and stained with hematoxylin and eosin (H&E) and with Periodic acid–Schiff (PAS). Crypt-cell proliferation and tight-junction were determined by immunohistochemistry with anti-Ki67 antibody (#652402, Biolegend, San Diego, CA) and anti-ZO-1 antibody (#GTX108592, Genetex, Irvine, CA), respectively. Apoptosis was detected by TUNEL assays, using the In Situ Cell-Death Detection Kit, peroxidase (POD) (Roche, Basel, Switzerland). For Alcian blue/PAS staining, the distal colon was harvested, submerged in Carnoy’s solution (Wako, Japan) at 4 °C for 2 h, and placed into 100% ethanol. The fixed colon tissues were embedded in paraffin, cut into 5 μm sections, and stained with Alcian blue/PAS. Microscopic images of the organoid cultures were obtained with a BZ-X700 (Keyence, Osaka, Japan).

### Colonic epithelial cell preparation and flow cytometry

Mouse colonic epithelial cells were obtained as described previously^35^. For sorting experiments, single cell suspensions were prepared as described previously with slight modifications^36^. Pellets of isolated crypts were suspended with 2 ml/mouse of TrypLE Express (Invitrogen, Carlsbad, CA) and incubated for 25 min at 37 °C. The suspension of single epithelial cells was filtered through a 70-μM nylon mesh, washed with cold PBS containing 10% FBS, then pelleted by centrifugation at 440×*g* for 5 min. The cell suspension was stained with APC-conjugated anti-EpCAM antibody (#118,214, Biolegend, San Diego, CA). Dead cells were removed by propidium-iodide staining. Cells were sorted on a MoFlo Legacy Cell Sorter (Beckman Coulter Inc., Brea, CA).

### Organoid culture

Organoids were cultured as described previously with slight modifications^37^. Sorted EpCAM^+^ cells were mixed with Matrigel (Corning, Corning, NY) containing 750 ng/mL epidermal growth factor (Peprotech, Rocky Hill, NJ), 1.5 μg/mL Noggin (Miltenyi Biotec, Germany), and 15 μmol/L Jagged-1 (Anaspec, Fremont, CA). The cells were seeded on 96-well plates at 3,000 cells/10 μL of Matrigel per well. After the Matrigel polymerized, 100 μL of culture medium consisting of Advanced Dulbecco’s Modified Eagle Medium/F12 supplemented with 100 U/mL penicillin/streptomycin, GlutaMAX, 10 mmol/L HEPES, 1 × N2, 1 × B27, 1 mmol/L N-acetylcysteine, 2.5 μmol/L Thiazovivin (Cayman, Ann Arbor, MI), and 2.5 μmol/L CHIR99021 (Axon Medchem, The Netherlands) was added to each well. On day 2 of culture, 50 μL of culture medium containing 10% (v/v) R-spondin 1 culture supernatant and growth factors (1 μM Jagged-1, 50 ng/mL epidermal growth factor, and 100 ng/mL Noggin at final concentration) was added to each well. On day 6, the number of organoids was counted and photographed in each well. The R-spondin 1 culture supernatant was harvested from the 293T-HA-RspoI-Fc cell line, which was provided by Calvin Kuo of Stanford University.

### Confocal imaging

Mouse colons were opened longitudinally, fixed with 10% (w/v) Formaldehyde Neutral Buffer Solution (Nacalai, Kyoto, Japan) for 30 min, and washed in PBS. The tissue was then transferred to a microslide glass with the villus side down and sealed with a microcover glass. Confocal images were obtained with a TCS SP8 (Leica Microsystems GmbH, Wetzlar, Germany).

### Quantitative RT-PCR

To determine gene expression levels, total RNA was extracted from EpCAM^+^ cells using the RNeasy Mini Kit (Qiagen, Germany), and cDNA was synthesized using random primers and SuperScript III Reverse Transcriptase (Thermo Fisher Scientific, Waltham, MA) according to the manufacturer’s instructions. The following primer sets were used: *Hprt,* forward 5′-GACCTCTCGAAGTGTTGGATAC-3′ and reverse 5′-CTTGCG CTCATCTTAGGCT-3′; *Irf2*, forward 5′-ACTGGGCGATCCATACAGGAA-3′ and reverse 5′-GTAGACTCTGAAGGCGTTGTTT-3′. *Ifit2*, forward 5′-AGTACAACGAGTAAGGAGTCACT-3′ and reverse 5′-AGGCCAGTATGTTGCACATGG-3′; *Ifitm3,* forward 5′-CCCCCAAACTACGAAAGAATCA-3′ and reverse 5′-ACCATCTTCCGATCCCTAGAC-3′; *Oas1g,* forward 5′-CTGCATCAGGAGGTGGAGTT-3′ and reverse 5′-ATGAGGATGGTGTAGATTAAGGG-3′; and *Stat1,* forward 5′-TCACAGTGGTTCGAGCTTCAG-3′ and reverse 5′-CGAGACATCATAGGCAGCGTG-3′.

### BrdU incorporation assay

BrdU (Sigma, St. Louis, MO) was intraperitoneally injected into mice (100 mg/kg body weight). After 2 and 48 h, the colons were fixed, embedded in paraffin, and then stained with a rat anti-BrdU antibody (#NB500-169, Novus Biologicals, Littleton, CO), biotin-conjugated anti-rat IgG (#13-4813-85, Invitrogen), and streptavidin-HRP (Zymed Laboratories Inc., San Francisco, CA). Epithelial migration was determined as previously described with slight modification^38^. The migration rate was determined as the distance of a BrdU-labeled cell from the crypt bottom divided by the total depth of the crypt. Distances were measured using ImageJ software (NIH).

### Fecal DNA extraction

Feces were collected and stored at − 80 °C until ready for use. Fecal DNA was extracted as previously described with minor modifications^39^. Briefly, 10 mg feces were suspended using sterilized sticks in 300 μl of TE10. The fecal suspension was incubated with 15 mg/ml lysozyme (Wako, Osaka, Japan) at 37 °C for 1 h, after which purified achromopeptidase (Wako) was added to a final concentration of 2000 units/ml, and the suspension was incubated at 37 °C for 30 min. Next, 1% (w/v) sodium dodecyl sulfate and 1 mg/ml proteinase K (Merck Japan) was added, and the suspension was incubated at 55 °C for 1 h and then centrifuged. The bacterial DNA was purified using a phenol/chloroform/isoamyl alcohol (25:24:1) solution and precipitated by adding ethanol and sodium acetate. The DNA was treated with RNase and precipitated with polyethylene glycol.

### Microbiota analysis by 16S rRNA sequencing

The V4 variable region (515F–806R) of 16S rRNA was sequenced on an Illumina Miseq as described by Kozich et al.^40^*.* Each reaction mixture contained 15 pmol of each primer, 0.2 mM deoxyribonucleoside triphosphates, 5 μl of 10 × Ex Taq HS buffer, 1.25 U Ex Taq HS polymerase (Takara Bio, Inc., Shiga, Japan), 50 ng extracted DNA, and sterilized water to reach a final volume of 50 μl. PCR conditions were as follows: 95 °C for 2 min and then 25 cycles of 95 °C for 20 s, 55 °C for 15 s, and 72 °C for 5 min, followed by 72 °C for 10 min. The PCR product was purified by AMPure XP (Beckman Coulter, Inc., Brea, CA) and quantified using a Quant-iT PicoGreen dsDNA Assay Kit (Life Technologies Japan, Ltd, Tokyo, Japan). Mixed samples were prepared by pooling approximately equal amounts of PCR amplicons from each sample. The pooled library was analyzed with an Agilent High-Sensitivity DNA Kit on an Agilent 2100 Bioanalyzer (Agilent Technologies, Santa Clara, CA) and quantified by real-time PCR using a KAPA Library Quantification Kit for Illumina (Roche, Basel, Switzerland) following the manufacturer’s protocols. The sample library was denatured and diluted based on quantification results. A sample library with 20% denatured PhiX spike-in was sequenced by Miseq using a 500-cycle kit. We obtained 2 × 250-bp paired-end reads.

We assigned taxonomy and estimated the relative abundance of sequencing data with the analysis pipeline of the QIIME software package^41^, and detected chimeras with UCHIME^42^. An operational taxonomic unit (OTU) was defined at 97% similarity. OTU taxonomy was assigned by comparison with the Greengenes database using RDPclassifier^43,44^. We summarized the proportions of the identified taxa in each sample and calculated the amount of bacterial diversity. Similarity between samples was calculated using Bray–Curtis distances as implemented in the R software (https://www.R-project.org/), and beta-diversity was visualized using principal coordinate analysis plots. Rarefaction curves were generated for each sample using rarefactions ranging from 10 to 5,010 reads, with 10 iterations of each calculation.

### Microarray analysis and gene set enrichment analysis (GSEA)

For microarray analysis, EpCAM^+^ cells sampled from the colon were analyzed by Agilent Expression array. GSEA was performed using microarray data with GSEA v2.0.13 software (Broad Institute). Gene sets were obtained from the Molecular Signatures Database (MSigDB) v4.0 distributed at the GSEA website (https://www.broadinstitute.org/gsea/msigdb/index.jsp). Pre-ranked lists of the mean expression changes and input signatures were derived from published microarray data^19,45^. The number of permutations was set to 1,000. Gene sets with a nominal *P* value < 0.05 were considered statistically significant. The microarray data are available in the GEO database (Accession number: GSE107142).

### Statistical analysis

The statistical significance of the obtained values was analyzed by Student’s two-tailed *t* test. A* P* value < 0.05 was considered significant.

## Supplementary information

Supplementary Information.
