# Supplementary material for: IRF2 maintains the stemness of colonic stem cells by limiting physiological stress from interferon
Source: Sci Rep. 2020 Sep 8;10:14639. doi: 10.1038/s41598-020-71633-3 (PMC7479133; doi:10.1038/s41598-020-71633-3)
Supplement: Supplementary file 1 — Supplementary Information. [file 41598_2020_71633_MOESM1_ESM.pdf]

IRF2 maintains the stemness of colonic stem cells by limiting physiological stress from interferon

Kana Minamide[1,4], Taku Sato[1,2,4], Yusuke Nakanishi[1], Hiroshi Ohno[3], Tamotsu Kato[3], Jumpei Asano[1], Toshiaki Ohteki[1,5]*

[1]Department of Biodefense Research, Medical Research Institute, Tokyo Medical and Dental University (TMDU), Tokyo, Japan.

[2]Japan Science and Technology Agency, Precursory Research for Embryonic Science and Technology (PRESTO), Tokyo, Japan

[3]Laboratory for Intestinal Ecosystem, RIKEN Center for Integrative Medical Sciences (IMS), Kanagawa, Japan.

[4]These authors contributed equally: Kana Minamide, Taku Sato

[5]Lead contact.

*Correspondence: ohteki.bre@mri.tmd.ac.jp

Supplementary Figure S1. Minamide et al.

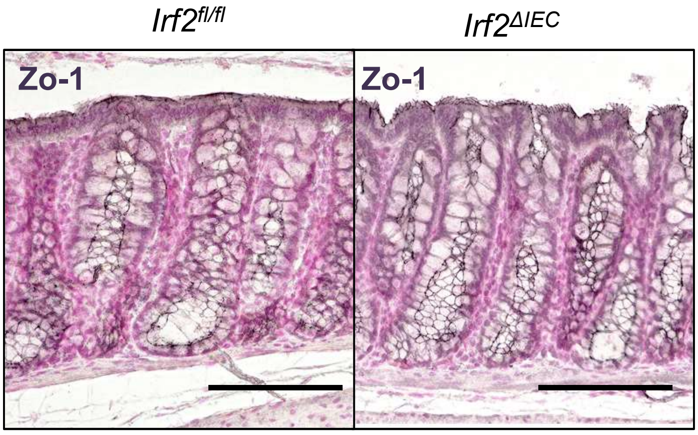

Figure S1. Related to Figure 2.
Representative images of ZO-1 staining of the distal colon tissue from *Irf2*$^{fl/fl}$ and *Irf2*$^{\Delta IEC}$ mice (n=2). Scale bars: 100 µm.

Supplementary Figure S2. Minamide et al.

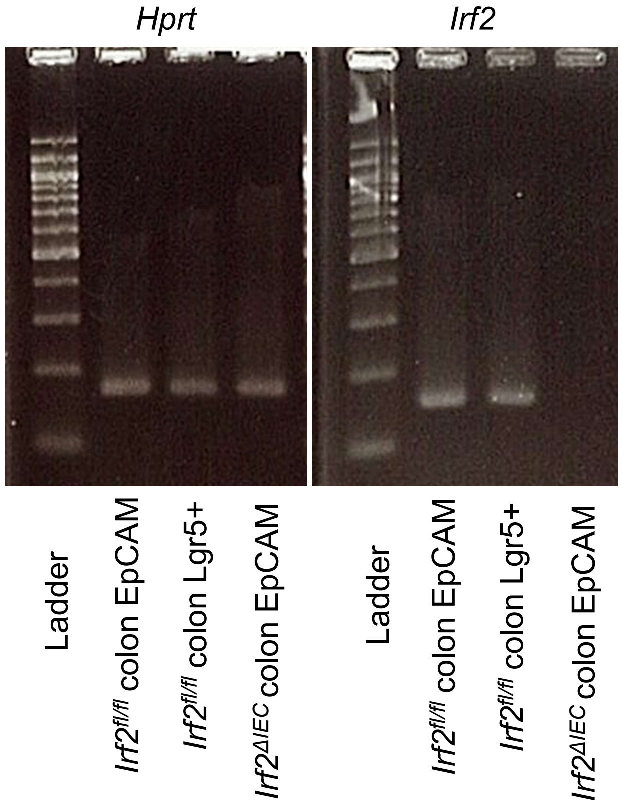

Figure S2. Related to Figure 3.
RT-PCR of *Irf2* mRNA in sorted EpCAM^{+} cells from $Irf2^{fl/fl}$-$Lgr5^{GFP}$ and $Irf2^{\Delta IEC}$-$Lgr5^{GFP}$ mice, and sorted Lgr5-GFP-positive cells from $Irf2^{fl/fl}$-$Lgr5^{GFP}$ mice. No *Irf2* mRNA was detected in the colon EpCAM^{+} cells of $Irf2^{\Delta IEC}$-$Lgr5^{GFP}$ mice.

Supplementary Figure S3. Minamide et al.

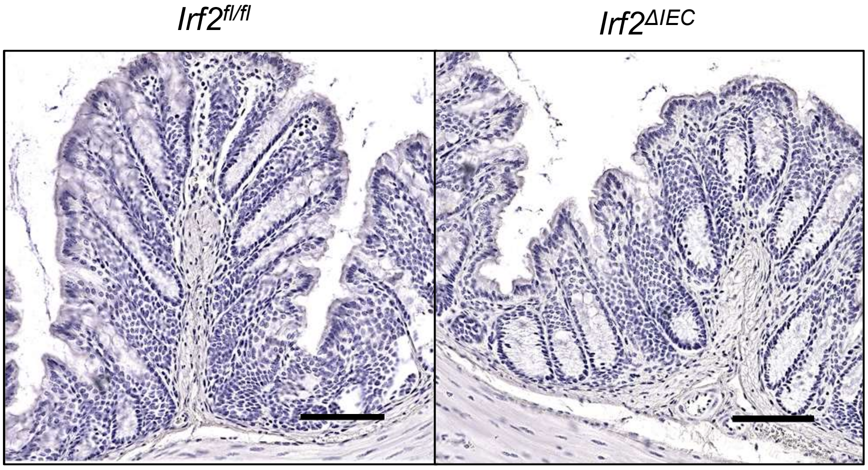

Figure S3. Related to Figure 3.
Representative images of TUNEL staining in the colon of naïve *Irf2*$^{fl/fl}$ and *Irf2*$^{\Delta IEC}$ mice (n=2). Scale bars: 100 µm.

Supplementary Figure S4. Minamide et al.

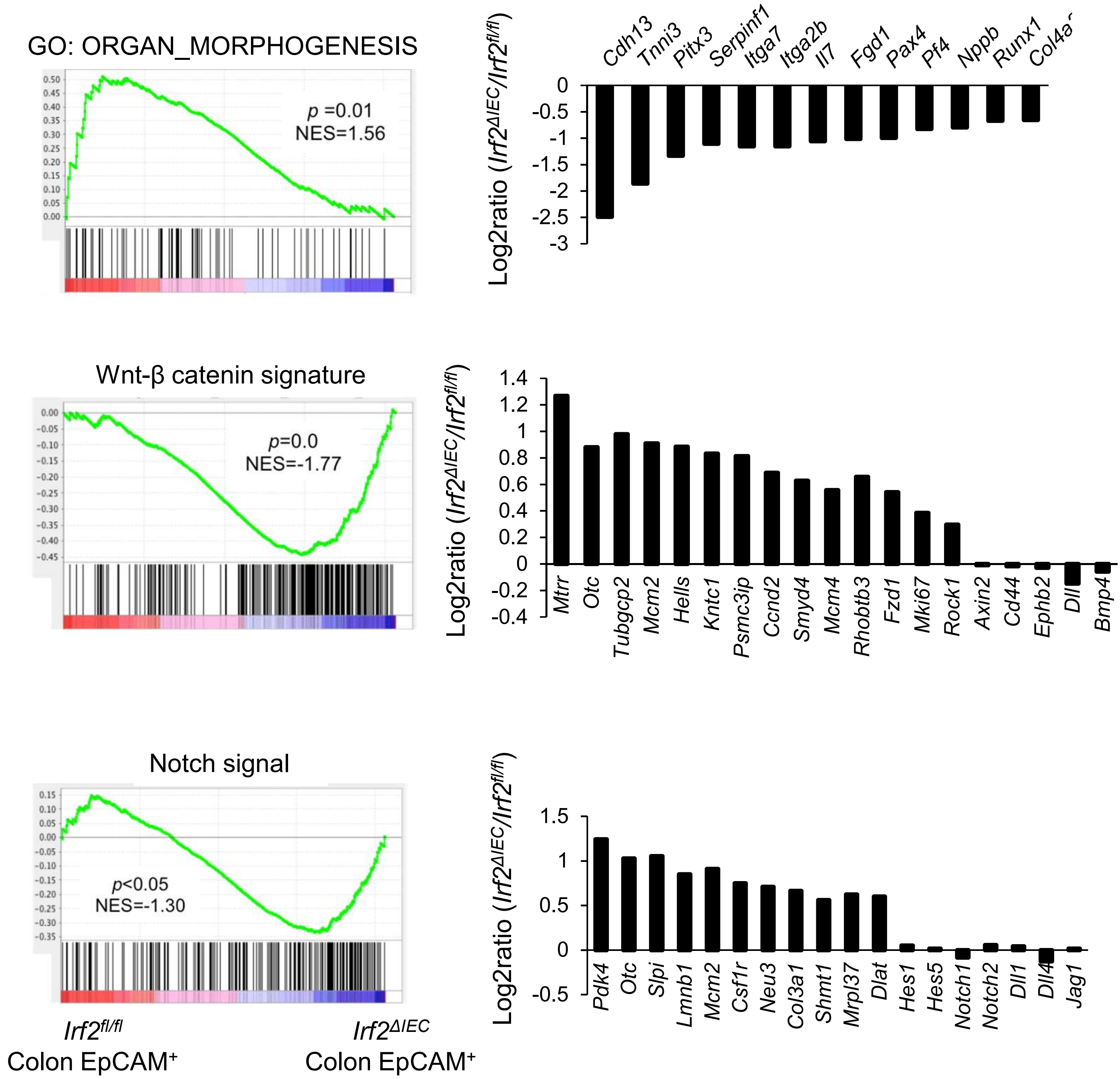

Figure S4. Related to Figure 4.

Gene set enrichment analysis and bar graphs showing the relative expression of typical genes for each signature in *Irf2*$^{\Delta IEC}$ compared to *Irf2*$^{fl/fl}$ mice; Upper: C5 (GO gene sets), middle: Wnt-β catenin signaling (GSE8818), and lower: notch signaling (GSE66751). Results are depicted as the log2 ratio determined by microarray.

Supplementary Figure S5. Minamide et al.

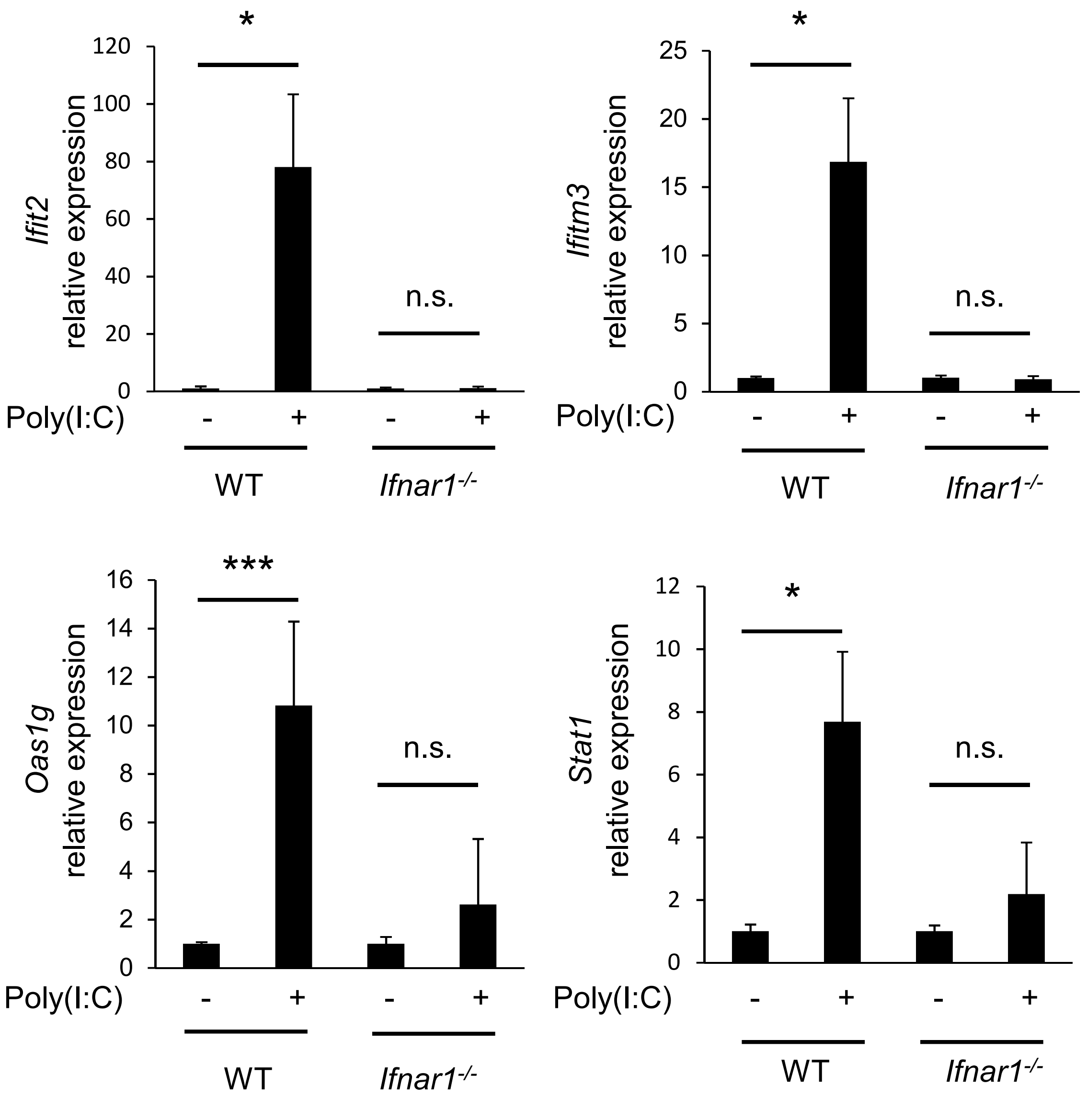

Figure S5. Related to Figure 5.
Relative mRNA expressions of IFN-inducible genes in the colon epithelial cells of the indicated groups. The results were normalized to the *Hprt* expression. Data represent mean ± SD; n=3 for each group. *$P < 0.05$, ***$P < 0.001$ by Student's *t* test. n.s., not significant.

Supplementary Figure S6. Minamide et al.

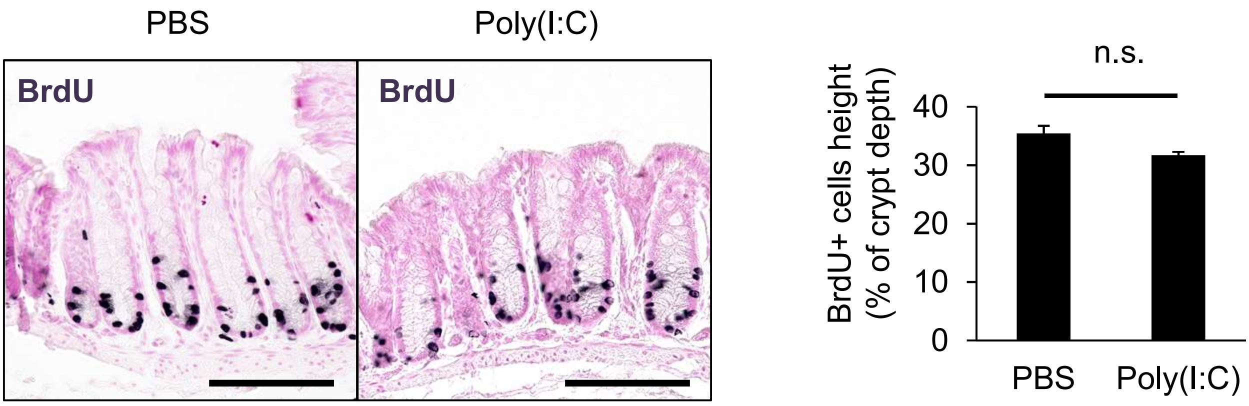

Figure S6. Related to Figure 5.
Representative images of BrdU-labeled cells in the colon of WT mice treated with either PBS or poly(I:C). The position of BrdU-labeled cells 2 hours after BrdU injection was determined. At least 50 crypts per mouse were analyzed. Data represent mean ± SD for 3 mice. Scale bars: 100 µm. Statistical analysis was performed by Student's *t* test. n.s., not significant.
